# Toll-Like Receptor 4 Mediates Inflammatory Cytokine Secretion in Smooth Muscle Cells Induced by Oxidized Low-Density Lipoprotein

**DOI:** 10.1371/journal.pone.0095935

**Published:** 2014-04-22

**Authors:** Ke Yang, Xiao Jie Zhang, Li Juan Cao, Xin He Liu, Zhu Hui Liu, Xiao Qun Wang, Qiu Jin Chen, Lin Lu, Wei Feng Shen, Yan Liu

**Affiliations:** 1 Department of Cardiology, Rui Jin Hospital, Medical School of Jiaotong University, Shanghai, People’s Republic of China; 2 Institute of Cardiovascular Diseases, Medical School of Jiaotong University, Shanghai, People’s Republic of China; Scuola Superiore Sant’Anna, Italy

## Abstract

Oxidized low-density lipoprotein (oxLDL)-regulated secretion of inflammatory cytokines in smooth muscle cells (SMCs) is regarded as an important step in the progression of atherosclerosis; however, its underlying mechanism remains unclear. This study investigated the role of toll-like receptor 4 (TLR4) in oxLDL-induced expression of inflammatory cytokines in SMCs both *in vivo* and *in vitro*. We found that the levels of TLR4, interleukin 1-β (IL1-β), tumor necrosis factor-α (TNFα), monocyte chemoattractant protein 1 (MCP-1) and matrix metalloproteinase-2 (MMP-2) expression were increased in the SMCs of atherosclerotic plaques in patients with femoral artery stenosis. In cultured primary arterial SMCs from wild type mice, oxLDL caused dose- and time-dependent increase in the expression levels of TLR4 and cytokines. These effects were significantly weakened in arterial SMCs derived from TLR4 knockout mice (TLR4−/−). Moreover, the secretion of inflammatory cytokines was blocked by TLR4-specific antibodies in primary SMCs. Ox-LDL induced activation of p38 and NFκB was also inhibited in TLR4−/− primary SMCs or when treated with TLR4-specific antibodies. These results demonstrated that TLR4 is a crucial mediator in oxLDL-induced inflammatory cytokine expression and secretion, and p38 and NFκB activation.

## Introduction

Atherosclerosis is a chronic inflammatory and fibro-proliferative disease [1]. It is accelerated by high lipid levels in the serum, and is correlated with future risk for symptomatic cardiovascular disease [2]. Low-density lipoprotein oxidation in the vascular wall is the main characteristic of atherosclerosis [3]. Oxidized low-density lipoprotein (oxLDL) contributes to both initiation and progression of atherosclerosis [4]–[6]. Previously studies found that oxLDL promoted the inflammatory response of monocyte-derived macrophages and endothelial dysfunction [7], [8]. Furthermore, smooth muscle cells (SMCs) are the main cell type in intimal thickenings and play a vital role in human atherosclerosis, as monocyte-derived macrophages and SMCs accumulate excess lipids and participate in the total intimal foam cell population [9].

Atherosclerosis involves the release of inflammatory cytokines, such as interleukin 1-β (IL1-β) and tumor necrosis factor-α (TNFα) which participate in pro-inflammatory signaling [10], [11]. Monocyte chemoattractant protein 1 (MCP-1) which can recruit circulating monocytes and T-cells to the site of activation and contribute to vascular inflammation [12]–[15] and production of which can be further induced by oxLDL [16]. Matrix metalloproteinase-2 (MMP-2), a major MMP derived from SMCs, is up-regulated and activated in human atherosclerotic lesions [17]–[19]. Therefore, inflammatory responses participate in all stages of atherosclerosis and promote its pathophysiological progression [20].

Toll-like receptor 4 (TLR4) is expressed in a variety of cells, such as the endothelial cells and smooth muscle cells [21], [22], and participates in the inflammatory response in atherosclerosis [23]. OxLDL induced inflammatory response involves many mechanisms and resent study found TLR4 is expressed in lipid-rich atherosclerotic plaques [24]. The extent of atherosclerosis can be notably decreased in TLR4 deficiency mice, suggesting a direct role of TLR4 as one of oxLDL receptors in oxLDL-induce inflammatory response and in the pathophysiology of atherosclerosis [25], [26].

SMCs are important for the development and stability of atherosclerosis lesions [27]. Using immunohistochemistry, we show in this study that activated TLR4 and inflammatory cytokines are co-localized in SMCs in human atherosclerotic plaques and that TLR4 plays a key role in inflammatory response mediated by oxLDL activation in SMCs. TLR4 is essential for oxLDL-induced IL-1β, TNF-α, MCP-1 and MMP-2 secretion in artery SMCs. These results provide a novel mechanism for atherosclerosis progression and advance our understanding of coronary artery disease.

## Materials and Methods

### Reagents and Antibodies

OxLDL (oxLDL, Serotec, UK) was used to stimulate smooth muscle cells. Immunohistochemical and western-blot antibodies used to detect TLR4, α-SMA, IL-1β, TNF-α, MCP-1 and MMP-2 were purchased from Abcam (MA, USA). 3, 3′ -Diaminobenzidine Liquid Substrate System was purchased from Sigma-Aldrich (MO, USA). Fetal bovine serum (FBS), F12: DMEM culture medium, penicillin and streptomycin were from Gibco BRL (Carlsbad, USA). The primary antibodies used were TLR4 (Abcam, USA), β-actin, NF-κB, phosphorylated-NF-κB, p38MAPK, phosphorylated-p38MAPK (p-p38MAPK), extracellular signal-regulated kinase 1/2 (ERK1/2), p-ERK1/2, c-Jun N-terninal kinase (JNK) and phosphorylated-JNK (Cell Signaling, MA, USA). HRP-conjunct antibody, Alexa 549- or Alexa 488- conjugated antibody (Cell Signaling, USA) was used as secondary antibody. Activation of TLR4 was blocked by Anti-mouse TLR4/MD-2 antibody (eBioscience, 16–9924, USA) and normal mouse IgG (eBioscience, USA) was used as negative control. Interleukin 1-β (IL-1β), tumor necrosis factor-α (TNF-α), monocyte chemoattractant protein 1 (MCP-1) and matrix metalloproteinase-2 (MMP-2) ELISA kits were obtained from R&D Systems (R&D, USA).

### Clinical Samples

Human femoral arteries with angiographic atherosclerotic plaques (stenosis >70%; 3 men; age, 70±3; 66.7% hypertensive; non-diabetic; 100% dyslipidemic) were obtained from 3 patients who underwent leg amputation. The internal thoracic arteries (n = 3) were obtained as normal artery. The study protocol was approved by the Ethics Committee of Rui Jin Hospital, Shanghai Jiaotong University School of Medicine, and written informed consent was obtained from all patients.

### Primary Smooth Muscle Cells Culture

Wild-type (C57BL/6) and TLR4 knockout mice (TLR4^−/−^) (male, 6 weeks old) were obtained from the Model Animal Research Center of Nanjing University (Nanjing, China). Animals were fed for 2 weeks and kept on a 12 h light/12 h dark cycle. All animals were sacrificed and arteries were gathered to culture the primary smooth muscle cells (SMCs). The cells were grown in F12: DMEM (1∶1) supplemented with 20% fetal bovine serum, monothioglycerol (1.2 mmol/L), L-glutamine (2 mmol/L), penicillin (100 U/mL) and streptomycin (100 µg/mL). Cells were grown with 5% CO_2_ at 37°C. The animal experimental protocol complies with the Animal Management Rules of the Chinese Ministry of Health (document No 55, 2001) and was approved by the Animal Care Committee of Shanghai Jiaotong University.

### Immunohistochemistry

The human femoral arteries (n = 3) were used for histological and immunochemical analysis. Samples were fixed in 4% paraformaldehyde overnight, and cut into serial cryosections (5 µm thickness). Sections were used for hematoxylin and eosin (H&E) staining or immunohistochemistry analysis with the following antibodies: anti-αSMA (1∶50), anti-TLR4 (1∶50) and anti-IL-1β (1∶50), TNFα (1∶50), MCP-1 (1∶50), and MMP2 (1∶50). After incubation with horseradish peroxidase (HRP) -conjugated secondary antibodies (1∶100), sections were incubated with 3, 3′ -Diaminobenzidine.

The sections were used for immunofluorescence analysis. The tissues were immunostained with anti-TLR4 (1∶50) or anti-αSMA antibody (1∶50) for 12 h at 4°C and incubated with Alex 549- or Alexa 488- conjugated secondary antibody (1∶1000).

### Blocking TLR4 Activation

C57BL/6 mice primary SMCs were seeded into 6-well plates at a density of 5×10^6^ cells/well, and then pretreated with anti-TLR4 antibody (5 ug/mL) or mouse IgG (5 ug/mL) as negative control for 1 h and subsequently stimulated with oxLDL.

### Measurement of Inflammatory Cytokines in Conditioned Medium

The supernatant of primary SMCs was collected after stimulation, and levels of IL-1β, TNFα, MCP-1 and MMP2 were measured using commercially available ELISA kits.

### Western Blot

Cells were lysed with the ProteoJET Mammalian Cell Lysis Reagent (Fermentas, MD, USA) to extract cytoplamic proteins. Equal amounts of protein extracts were subjected to 12% SDS/PAGE and blotted onto a poly (vinylidenedifluoride) membrane. The membrane was blocked and probed overnight at 4°C with antibodies against TLR4 (1∶500), β-actin (1∶2000), total p38 (1∶1000), phosphorylated p38 (1∶1000), total NF-κB (1∶1000), phosphorylated NF-κB (1∶1000), total ERK1/2 (1∶1000), phosphorylated ERK1/2 (1∶1000), total JNK (1∶1000),phosphorylated JNK (1∶1000), IL-1β (1∶1000), TNFα (1∶1000), MCP-1 (1∶1000), and MMP2 (1∶1000) followed by incubation with horseradish peroxidase-conjugated secondary antibodies (1∶5000) for 1 h at room temperature. Blots were developed using an ECL detection system (Millipore, MA, USA). Each image was captured and the intensity of each band was analyzed with Quantity One (Bio-Rad).

### Quantitative Real-time RT-PCR

Total RNA was extracted as described above. Briefly, 5 ug of total RNA was reverse-transcribed into cDNA using a reverse transcription system (Promega, WI, USA). PCR amplification was performed with Power SYBR Green PCR Master Mix (Applied BioSystems, CA, USA) in a StepOne (Applied BioSystems). The oligonucleotides used in quantitative real-time RT-PCR analysis are listed in Table 1. Gene expression levels were normalized with beta-actin, and data were analyzed with StepOne software v2.1 (Applied BioSystems).

**Table 1:** The primer has been used for Realtime-PCR.

Gene	Forward primer	Reverse primer	products size (bp)
TLR4	5′-AATCTGGTGGCTGTGGAG-3′	5′-CCCTGAAAGGCTTGGTCT-3′	287
IL-1β	5′-AATCTCGCAGCAGCACAT-3′	5′-CTTCTCCACAGCCACAAT-3′	67
TNF-a	5′-GCGGTGCCTATGTCTCA-3′	5′-CACTTGGTGGTTTGCTACG-3′	220
MCP-1	5′-TGGGTCCAGACATACATT-3′	5′-TACGGGTCAACTTCACAT-3′	121
MMP2	5′-CCCCGATGCTGATACTGA-3′	5′-CTGTCCGCCAAATAAACC-3′	152
β-actin	5′-CTGTCCCTGTATGCCTCTG-3′	5′-ATGTCACGCACGATTTCC-3′	218

### Statistical Analysis

All values are expressed as mean ±SD. Student’s paired t test was performed for comparison of paired samples, and ANOVA was used for multiple group comparisons, followed by Friedman’s posttest. A probability (*p*) value <0.05 was considered significant.

## Results

### Smooth Muscle Cells in Atherosclerotic Plaques Present Evident TLR4 Expression and Inflammatory Cytokines Secretion

**Figure 1:**
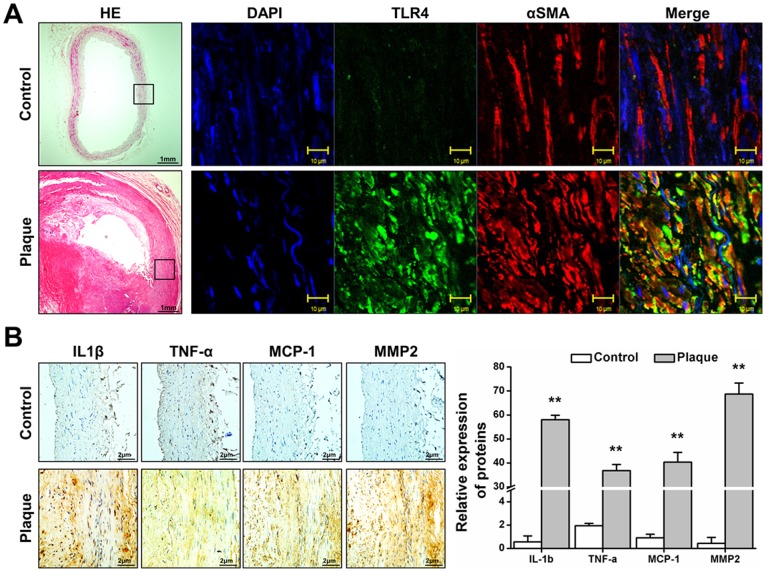
Expression of TLR4 and inflammatory cytokines in the SMCs of plaques. Human femoral arteries from patients with angiographic atherosclerotic plaques and interal thoracic artery without plaque were assessed by histological and immunochemical analysis. (A). Sections were stained with hematoxylin and eosin. Or immunofluorescence stains of αSMA and TLR4. (B) Immunohistochemical stains of IL-1β, TNFα, MCP-1 and MMP2; counterstained with hematoxylin. H&E is shown at 40×magnification, and immunofluorescence is shown at 1200× magnification B is shown at 200× magnification. Results are representative of 3 independent experiments. The expression levels of IL-1β, TNF-α, MCP-1 and MMP-2 detected by IHC were determined by assessing its staining using software image pro-plus 6.0. The results were showed as integrated optical density (IOD)/area. Three different sections and five different fields in each section have been detected. (n = 3, Mean±SD, **P<0.01 compared with control).

To investigate the pathology of atherosclerosis, femoral arteries from patients with femoral artery stenosis and internal thoracic artery (as control) were stained with hematoxylin and eosin. Routine pathological examination of human femoral artery plaques showed lipid deposition, macrophage rupture and vascular muscular thickening nearby the lipid core of plaque (**Fig. 1** **A**). Immunofluorescence analysis of sections showed TLR4 co-localized with α-SMA in some SMCs nearby the lipid core of plaque, but there are only α-SMA positive without TLR4 expression in control (**Fig. 1** **A**). The main inflammatory cytokines participating in atherosclerosis, IL-1β, TNF-α, MCP-1 and MMP-2, were detected in the plaque. Immunohistochemistry analysis of serial sections showed SMCs (α-SMA-positive cells) nearby the lipid core of atheroma expressed high levels of inflammatory cytokines (**Fig. 1** **B**). These results indicated that in atherosclerotic plagues, SMCs near the lipid core region over-express TLR4 and secret inflammatory cytokines.

### OxLDL Promotes TLR4 and Inflammatory Cytokines Expression

**Figure 2:**
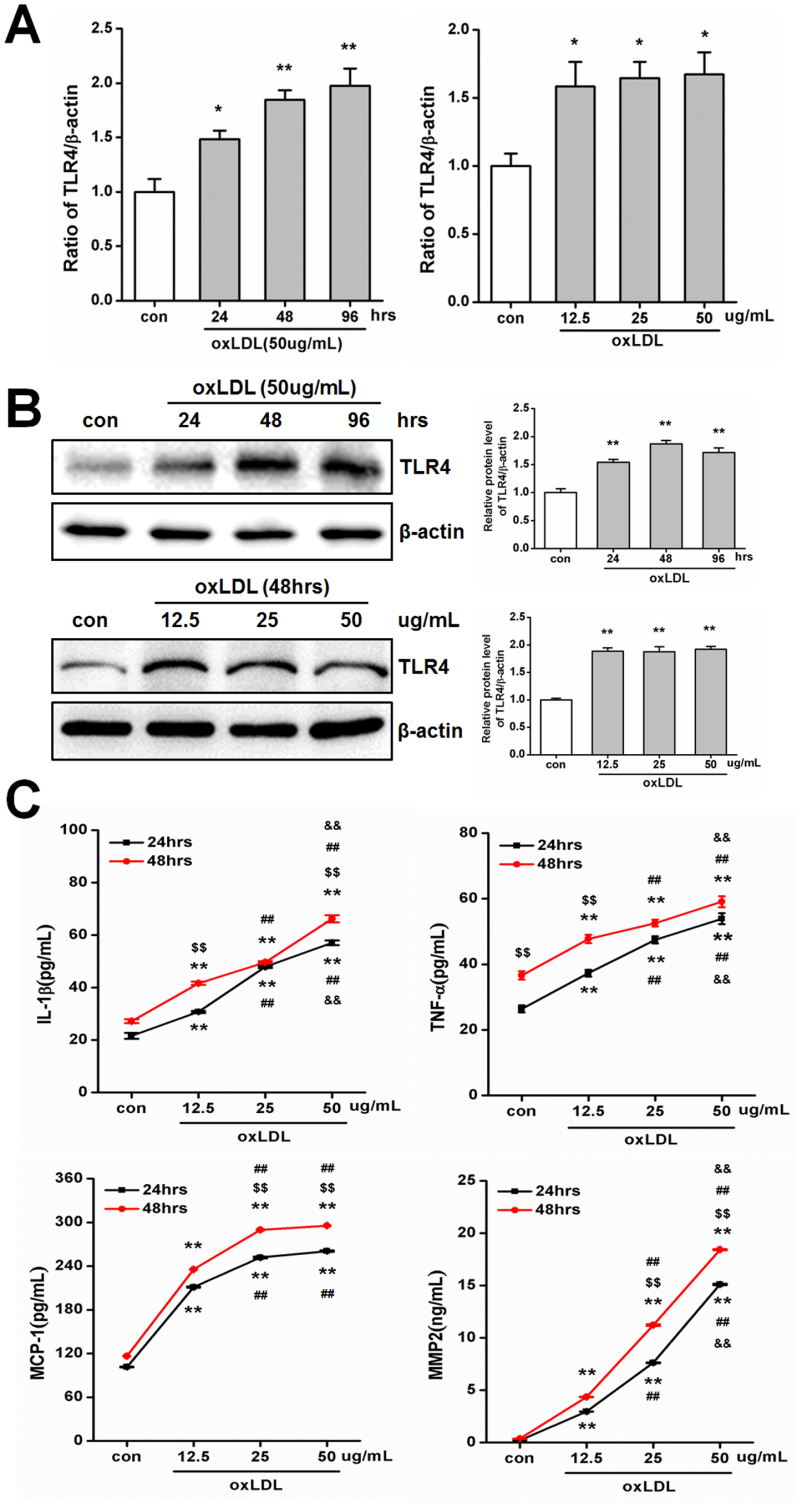
oxLDL promotes TLR4, IL-1β, TNF-α, MCP-1 and MMP-2 expression in primary smooth muscle cells. (A)–(B) Primary SMCs were incubated for increasing amount of time (24, 48 and 96 hrs with 50 ug/mL) and increasing doses (12.5, 25 and 50 ug/mL for 48 hrs) of oxLDL. The expression of TLR4 was detected by Realtime-PCR (A) and Western blotting (B) and quantified by densitometry in 3 independent experiments (A and B) as relative units (TLR4/β-actin). (Mean ± SD, n = 3; *P<0.05, **P<0.01 compared with con group). (C) oxLDL stimulated primary SMCs with increasing doses (12.5, 25 and 50 ug/mL for 24 and 48 hrs). Secretion of IL-1β, TNF-α, MCP-1 and MMP-2 was measured by ELISA and quantified in 3 independent experiments. (Mean ± SD, n = 3;**P<0.01 compared with con group, $$P<0.01 compared with 24 hrs treatment group, ##P<0.01 compared with 12.5 ug/mL oxLDL treatment group, && P<0.01 compared with 25 ug/mL oxLDL).

Based on these immunohistochemistry findings in atherosclerotic patients, we investigated whether TLR4 and inflammatory cytokines are involved in oxLDL-induced atherosclerosis model in mice. Primary SMCs were stimulated by oxLDL (for 24, 48 or 96 hrs and at a concentration of 12.5, 25 or 50 ug/mL) to assess its effects on TLR4 and inflammatory cytokines expression (**Fig. 2A–C**). Realtime PCR show the mRNA level of TLR4 is time-dependent increased (24 hours treatment = 1.48 fold, 48 hours treatment = 1.85 fold and 96 hours treatment = 1.97 fold vs. con, P<0.05, Fig. 2A) with 50 ug/mL oxLDL stimulated, however different dose of oxLDL increased TLR4 expression to same levels (12.5 ug/mL oxLDL = 1.58 fold, 25 ug/mL oxLDL = 1.65 fold and 50 ug/mL oxLDL = 1.67 fold vs. con, P<0.05, **Fig. 2A**). After 24 hrs of treatment with oxLDL at a concentration of 50 ug/mL, further increase in incubation time did not lead to a significant increase TLR4 protein expression (24 hours treatment = 1.54 fold, 48 hours treatment = 1.87 fold and 96 hours treatment = 1.72 fold vs. con, P<0.01, **Fig. 2B**), suggesting that induction of TLR4 by oxLDL is rapid. However, Expression of TLR4 was significantly increased after incubation with different doses of oxLDL for 48 hours (12.5 ug/mL oxLDL = 1.89 fold, 25 ug/mL oxLDL = 1.88 fold and 50 ug/mL oxLDL = 1.92 fold vs. con, P<0.01, **Fig. 2B**). The secretion of IL-1β, TNF-α, MCP-1 and MMP-2 were also promoted by oxLDL in primary SMCs (**Fig. 2C**). As well, a clear time- and dose-dependent increase was observed for most of these cytokines (**Fig. 2C**). The expression levels of intracellular is same as secreted after incubated with different dose and time of oxLDL (**Fig. S1**). These data indicated that in primary SMCs, oxLDL upregulates TLR4 and inflammatory cytokines in a time- and dose-dependent manner, and suggested that TLR4 levels are more sensitive to and more rapidly regulated by oxLDL stimulation.

### TLR4 Regulates oxLDL-dependent Secretion of IL-1β, TNF-α, MCP-1 and MMP-2 in Primary SMCs

**Figure 3:**
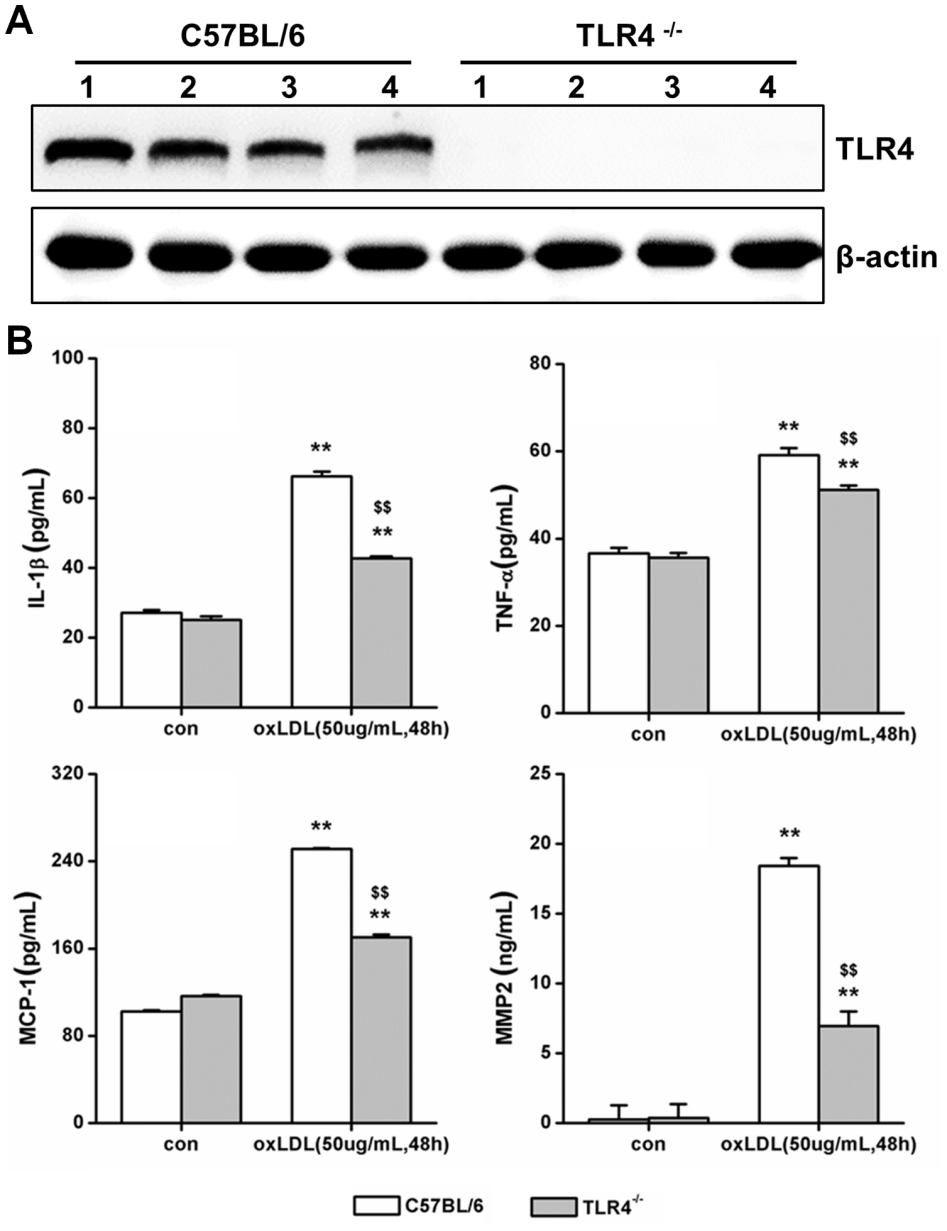
TLR4 knockout weakens IL-1β, TNF-α, MCP-1 and MMP-2 release in primary SMCs. The primary smooth muscle cells have been cultured from TLR4−/− and C57BL/6 mice. (A) The phenotype of TLR4−/− and C57BL/6 had been detected with Western-blot to assay TLR4 expression. Each type mice choose four different mice. (B) The cells have been incubated with or without oxLDL (50 ug/mL) for 48 hrs, and detected the level of IL-1β, TNF-α, MCP-1 and MMP-2 in the supernatant of cell culture medium. (Mean ± SD, n = 3;**P<0.01 compared with con group, $$P<0.01 compared with C57BL/6 mice group).

**Figure 4:**
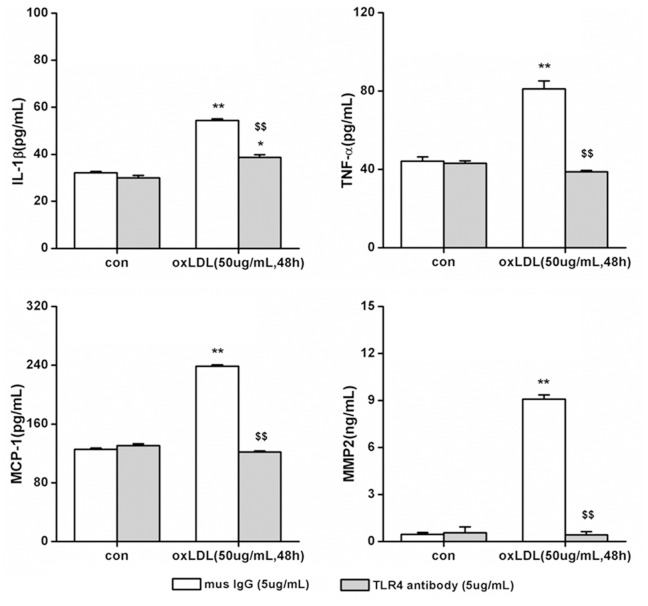
TLR4 blocking inhibits IL-1β, TNF-α, MCP-1 and MMP-2 release in primary SMCs. The primary smooth muscle cells have been pretreated with TLR4 blocking antibody, with mouse IgG used as control. The cells have been incubated with or without oxLDL (50 ug/mL) for 48 hrs, and levels of IL-1β, TNF-α, MCP-1 and MMP-2 in the supernatant of cell culture medium were measured. (Mean ± SD, n = 3; **P<0.01 compared with con group, $$P<0.01 compared with mouse IgG treatment group).

To test whether TLR4 signaling induced by oxLDL is important for inflammatory cytokines releasing in SMCs, we compared the responses in cytokine induction by oxLDL in primary SMCs cultured from the artery of either wild type (C57BL/6) or TLR4 knockout (TLR4^−/−^) mice. To identify the phenotype of wild type and TLR4−/−, expression of TLR4 had been detected, and it was null in TLR4^−/−^ SMCs compared with wild type (Fig. 3A). Primary SMCs were incubated with 50 ug/mL oxLDL for 48 hours. In wild type and TLR4^−/−^ SMCs, oxLDL treatment increased IL-1β, TNF-α, MCP-1 and MMP-2 secretion. However, compared with wild type SMCs, TLR4^−/−^ SMCs exhibited much less robust increase in the expression of IL-1β (TLR4^−/−^ = 42.76±0.52 pg/mL vs. C57BL/6 mice = 66.23±1.37 pg/mL, P<0.01), TNF-α (TLR4^−/−^ = 51.17±1.00 pg/mL vs. C57BL/6 mice = 59.11±1.66 pg/mL, P<0.01), MCP-1 (TLR4^−/−^ = 170.38±2.55 pg/mL vs. C57BL/6 mice = 251.40±0.85 pg/mL, P<0.01) and MMP-2 (TLR4^−/−^ = 6.96±1.04 pg/mL vs. C57BL/6 mice = 18.43±0.56 ng/mL, P<0.01) (**Fig. 3B**), suggesting that TLR4 plays an important role in ox-LDL mediated inflammatory cytokine release Further support for a critical involvement of TLR4 in this process was demonstrated by blocking the activation of TLR4 with TLR4-specific antibodies [28]. Compared with mouse IgG control, TLR4-specific antibodies inhibited the increase of oxLDL induced-cytokines completely (**Fig. 4** **, Fig. S2**). Taken together, these data showed that oxLDL-induced inflammatory cytokine secretion is significantly attenuated after TLR4 knockout and blocking, strongly suggesting that the ability of SMCs release IL-1β, TNF-α, MCP-1 and MMP-2 in response to oxLDL requires TLR4.

### OxLDL Activates P38 MAPK via TLR4 in Primary SMCs

**Figure 5:**
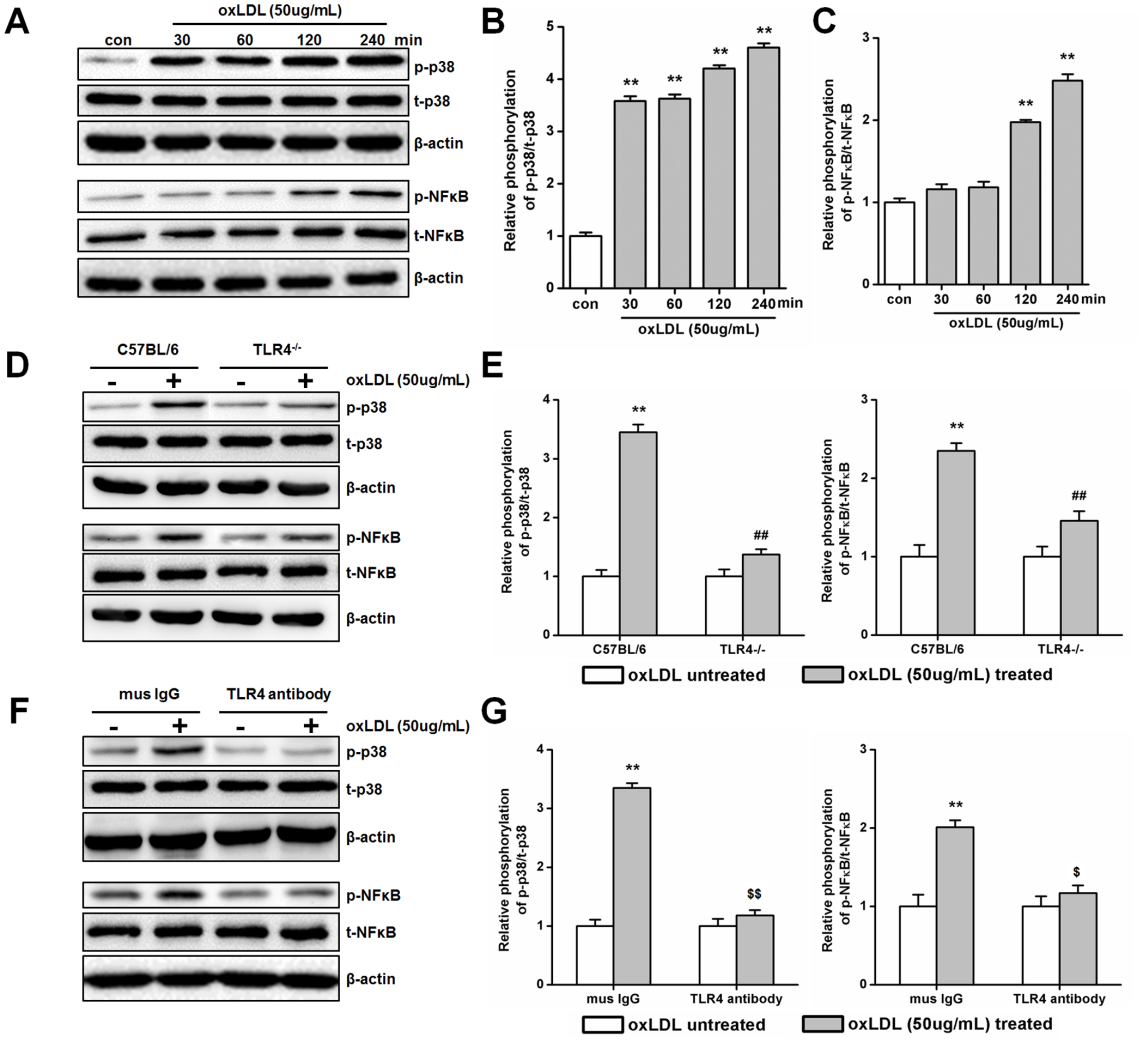
OxLDL induces TLR4-dependent activation of p38 MAPK in primary SMCs. (A)–(C) SMCs were incubated for increasing amount of time (30, 60,120 and 240 min with 50 ug/mL)of oxLDL. The phosphorylation of p38 and NF-κB was detected by Western blotting (A) and quantified by densitometry in 3 independent experiments (B and C) as relative units (p38 or NF-κB phosphorylated protein/total protein). (Mean ± SD, n = 3; *P<0.05, **P<0.01 compared with con group). (D)- (G) The primary SMCs of TLR4^−/−^ (C57BL/6 as control) and TLR4 antibody blocking (mouse IgG as control) have been treated with or without oxLDL (50 ug/mL) for 240 min to measure the activation of p38 or NF-κB in 3 independent experiments as relative units (p38 or NF-κB phosphorylated protein/total protein). (Mean ± SD, n = 3; **P<0.01 compared with oxLDL untreated group, ##P<0.01 compared with C57BL/6 group, $P<0.05, $$P<0.01 compared with mouse IgG group).

Finally, we hypothesized that TLR4 may interact with MAP kinases upon oxLDL treatment in SMCs [29], [30]. Phosphorylation of p38, ERK1/2 and JNK could be detected in culture SMCs. But only the phosphorylation of p38 was significantly increased after oxLDL treatment (at a concentration of 50 ug/ml, for 30, 60, 120 and 240 min) (**Fig. 5A–B** **and Fig. S3**). Interestingly, phosphorylation of NF-κB, as the downstream effect of p38 phosphorylation, was also stimulated after 120 min of oxLDL treatment (**Fig. 5A and C**). To determine whether oxLDL-induced activation of p38 MAPK is dependent on TLR4 in SMCs, TLR4 knockout primary SMCs or the blocking antibodies were used to reduce TLR4 expression levels and activity, respectively. Compared with control treatments, TLR4 knockout and blocking significantly decreased p38 and NF-κB activation in SMCs (**Fig. 5D–G**). These results suggest a mechanism by which TLR4 and p38 MAPK together mediate inflammatory response upon oxLDL treatment.

## Discussion

This study demonstrates that TLR4, IL-1β, TNF-α, MCP-1 and MMP-2 expression is increased in the smooth muscle cells near the lipid core region in atherosclerotic plaques, and that oxLDL and TLR4 interact to facilitate inflammatory secretion in the primary SMCs. This interaction is the important step in the development of atherosclerosis. Monocyte derived-macrophages have been regarded as mainly inflammatory response cells in the plaque in previous studies. During preparation of this manuscript, a recent publication showed that SMCs play an important role in the pathology of atherosclerosis. Kiyan et al [31] found that oxLDL induced SMCs releasing G-CSF and GM-CSF via urokinase receptor association with CD36 and TLR4, suggesting that SMCs participated in active modulation of macrophage functions. A major finding of our study is that TLR4 plays a crucial role in SMCs inflammatory response. TLR4 directly regulated oxLDL-induced inflammatory cytokines secretion in SMCs. We found that knockout or blocking TLR4 in smooth muscle cells significantly reduces IL-1β, TNF-α, MCP-1 and MMP-2 following oxLDL exposure, which mimics the pathophysiological conditions of atherogenesis in which SMCs transform into foam cells by taking up oxLDL and release inflammatory cytokines [32]. Furthermore, oxLDL induced inflammatory response and activation of p38 MAPK as TLR4 downstream signaling pathway in primary SMCs, but this effect was eliminated by TLR4 knockout or blocking. Thus our data emphasize the important role of TLR4 in functional interactions between these proteins in the inflammatory pathway.

It is likely that TLR4 signaling regulates SMCs inflammatory response via multiple mechanisms. First, Toll-like receptors (TLRs) are a family of surface molecules, and involved in innate immune responses and inflammatory responses, such as ischemic reperfusion injury and atherosclerosis [33], [34]. In all members of TLRs, TLR4 is the most relevant one with atherosclerosis [35], [36]. Some studies found that human TLR4 is expressed in murine and human lipid-rich atherosclerotic plaques, including areas infiltrated by macrophages, and oxLDL but not native LDL induces up-regulation of TLR4 expression in macrophages, suggesting that TLR4 has a potential role in lipid-mediated proinflammatory signaling in atherosclerosis [24], [37]. Second, the intracellular signaling induced by oxLDL mainly includes mitogen-activated protein kinases (MAPKs) pathways, including p38, JNK, ERK1/2, which play a critical role in regulating the inflammatory responses, proliferation and apoptosis in various cell types [38], [39], and are an important group of downstream of mediators in TLR4 signaling, and be activated after specific phosphorylation [40]. In our study, p38 and NF-kB were activated by oxLDL, which is specific inflammatory regulation model in SMCs. Thus, TLR4 could alter the expression and activation of a variety of genes involved in inflammatory response to affect cytokines contents.

Otherwise, wild type and TLR4−/− mice had been fed with normal or high fat diet for one month; the expression of cytokines had been detected. The immunochemical results show that inflammatory cytokines were increased in the group of wild type fed with high fat diet compared with other groups (P<0.01), but it has no-significant change in TLR4−/− (P>0.05, **Fig. S4**). At the same time, we compared the effect of oxLDL and LDL on cytokines expression in SMCs. The results show that the level of cytokines had been promoted with oxLDL and LDL stimulated. Compared with LDL effect on expression of cytokines, oxLDL further strengthened the affection of IL-1β, TNF-α, MCP-1 and MMP-2 (P<0.01, **Fig. S5**). The pathological process of atherosclerosis is a period of LDL from deposit to oxidize [41]. However, oxLDL exhibited more harmful than LDL in atherosclerosis has been confirmed [42]. Else wile, SMCs are mainly taking part in middle and late period of plaque formed. All of these results demonstrated that SMCs participated in chronic inflammatory response of atherosclerosis.

IL-1β, TNF-α, MCP-1 and MMP-2 can promote the progression of atherosclerosis. In this study, after incubated with oxLDL, the secretion of inflammatory cytokines rose at a dose- and time-dependent way in primary cultured SMCs. But this effect had been weakening by knockout TLR4 or use TLR4 antibody to block TLR4 pathway. Interestingly, compared with TLR4 antibody blocking, knockout TLR4 only weakens the inflammatory cytokines release. This illustrates that multiple mechanisms may participate in oxLDL induced inflammatory response in SMCs [31]. TLR4 knockout mimics a long-term to deprive the function of TLR4, which may trigger compensatory mechanism. On the contrary, TLR4 antibody only plays a short-term effect in blocking TLR4 activation. The long-term, compensatory mechanism may not be activated in such a scenario, so we could observe that the effect of TLR4 antibody inhibited IL-1β, TNF-α, MCP-1 and MMP-2 secretion more robustly than TLR4−/−.

In our results, we found that oxLDL (50 ug/mL) induced TLR4 protein expression in 48 and 96 hours, but it decreased in 96 hours. On the contrary, the mRNA level of TLR4 is higher after oxLDL stimulated 96 hours than 48 hours. There may possess a mechanism of negative feedback to regulate the protein not mRNA expression of TLR4. We will identify this affection in the further studies.

In summary, we demonstrated in this work the expression of TLR4 and inflammatory cytokines in SMCs in human atherosclerotic plaque. Using an in vitro model, we showed that this activation is functionally and physically associated with TLR4. The clinical implication of this study is that deactivation of TLR4 by humanized antibody could effectively attenuate the pathogenesis of atherosclerosis.

## Supporting Information

Figure S1 **oxLDL promotes IL-1β, TNF-α, MCP-1 and MMP-2 expression in cytoplasm of primary smooth muscle cells.** Primary SMCs were incubated for increasing amount of time (24, 48 and 96 hrs with 50 ug/mL) and increasing doses (12.5, 25 and 50 ug/mL for 48 hrs) of oxLDL. The expression of cytokines were detected by Western-blot (A and C) and quantified by densitometry in 3 independent experiments (B and D) as relative units (cytokines/β-actin). (Mean ± SD, n = 3; **P<0.01 compared with con group).
(TIF)

Figure S2 **The affection of TLR4 antibody blocks IL-1β, TNF-α, MCP-1 and MMP-2 expression in SMCs.** Different doses (0, 1.25, 2.5 and 5 ug/mL) of TLR4 antibody pretreated with SMCs for 1 hour and stimulated with oxLDL (50 ug/mL). The expressions of cytokines were detected by Realtime-PCR in 3 independent experiments as relative units (cytokines/β-actin). (Mean ± SD, n = 3; *P<0.05, **P<0.01 compared with antibody un-treatment group; #P<0.05, ##P<0.01 compared with 1.25 ug/mL antibody treatment group; $$P<0.01 compared with 2.5 ug/mL antibody treatment group).
(TIF)

Figure S3 **oxLDL has none effect on ERK1/2 and JNK in SMCs.** (A)–(C) SMCs were incubated for increasing amount of time (30, 60,120 and 240 min with 50 ug/mL)of oxLDL. The phosphorylation of JNK and ERK1/2 was detected by Western blotting (A) and quantified by densitometry in 3 independent experiments (B and C) as relative units (JNK or ERK1/2 phosphorylated protein/total protein). (Mean ± SD, n = 3).
(TIF)

Figure S4 **The expression of cytokines in the SMCs of artery of wild type (C57BL/6) and TLR4−/− mice fed with high fat or normal fat diet.** The wild type and TLR4−/− mice fed with high fat or normal fat diet for one month. (A) The αSMA had been used to identify the SMCs. In the αSMA-positive region, the expression levels of IL-1β, TNF-α, MCP-1 and MMP-2 detected by IHC were determined by assessing its staining using software image pro-plus 6.0. (B) The results were showed as integrated optical density (IOD)/area. Three different sections and five different fields in each section have been detected. (n = 3, Mean±SD, *P<0.05, **P<0.01 compared with normal fat diet fed group; ##P<0.01 compared with C57BL/6 group).
(TIF)

Figure S5 **LDL and oxLDL regulated IL-1β, TNF-α, MCP-1 and MMP-2 expression.** After LDL (50 ug/mL) or oxLDL (50 ug/mL) incubated with SMCs for 48 hours, expression of IL-1β, TNF-α, MCP-1 and MMP-2 had been test by Realtime-PCR. Un-treatment of LDL or oxLDL were used as control and normalized with β-actin. (Mean ± SD, n = 3, *P<0.05, **P<0.01 compared with CON; ##P<0.01 compared with LDL treatment group).
(TIF)
