# Supplementary figures and images for: Toll-Like Receptor 4 Mediates Inflammatory Cytokine Secretion in Smooth Muscle Cells Induced by Oxidized Low-Density Lipoprotein
Source: PLoS One. 2014 Apr 22;9(4):e95935. doi: 10.1371/journal.pone.0095935 (PMC3995878; doi:10.1371/journal.pone.0095935)

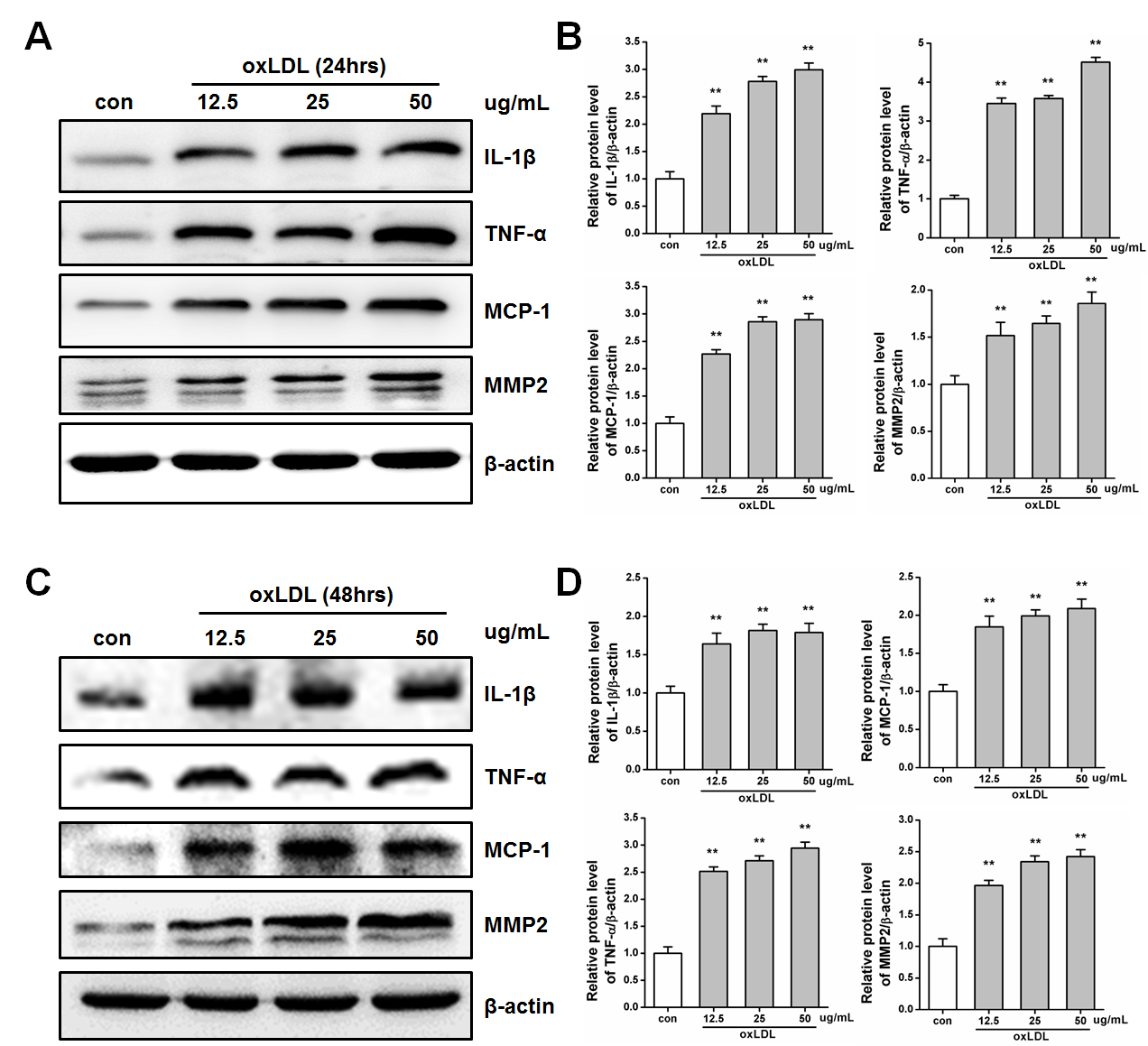

Supplement: Figure S1 — oxLDL promotes IL-1β, TNF-α, MCP-1 and MMP-2 expression in cytoplasm of primary smooth muscle cells. Primary SMCs were incubated for increasing amount of time (24, 48 and 96 hrs with 50 ug/mL) and increasing doses (12.5, 25 and 50 ug/mL for 48 hrs) of oxLDL. The expression of cytokines were detected by Western-blot (A and C) and quantified by densitometry in 3 independent experiments (B and D) as relative units (cytokines/β-actin). (Mean ± SD, n = 3; **P<0.01 compared with con group). (TIF) [file pone.0095935.s001.tif]

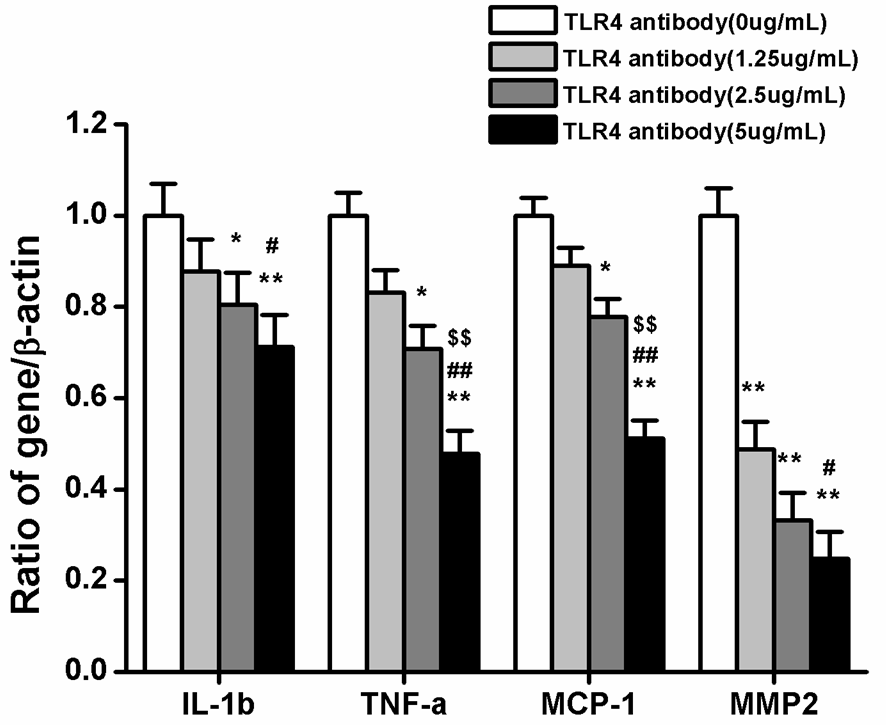

Supplement: Figure S2 — The affection of TLR4 antibody blocks IL-1β, TNF-α, MCP-1 and MMP-2 expression in SMCs. Different doses (0, 1.25, 2.5 and 5 ug/mL) of TLR4 antibody pretreated with SMCs for 1 hour and stimulated with oxLDL (50 ug/mL). The expressions of cytokines were detected by Realtime-PCR in 3 independent experiments as relative units (cytokines/β-actin). (Mean ± SD, n = 3; *P<0.05, **P<0.01 compared with antibody un-treatment group; #P<0.05, ##P<0.01 compared with 1.25 ug/mL antibody treatment group; $$P<0.01 compared with 2.5 ug/mL antibody treatment group). (TIF) [file pone.0095935.s002.tif]

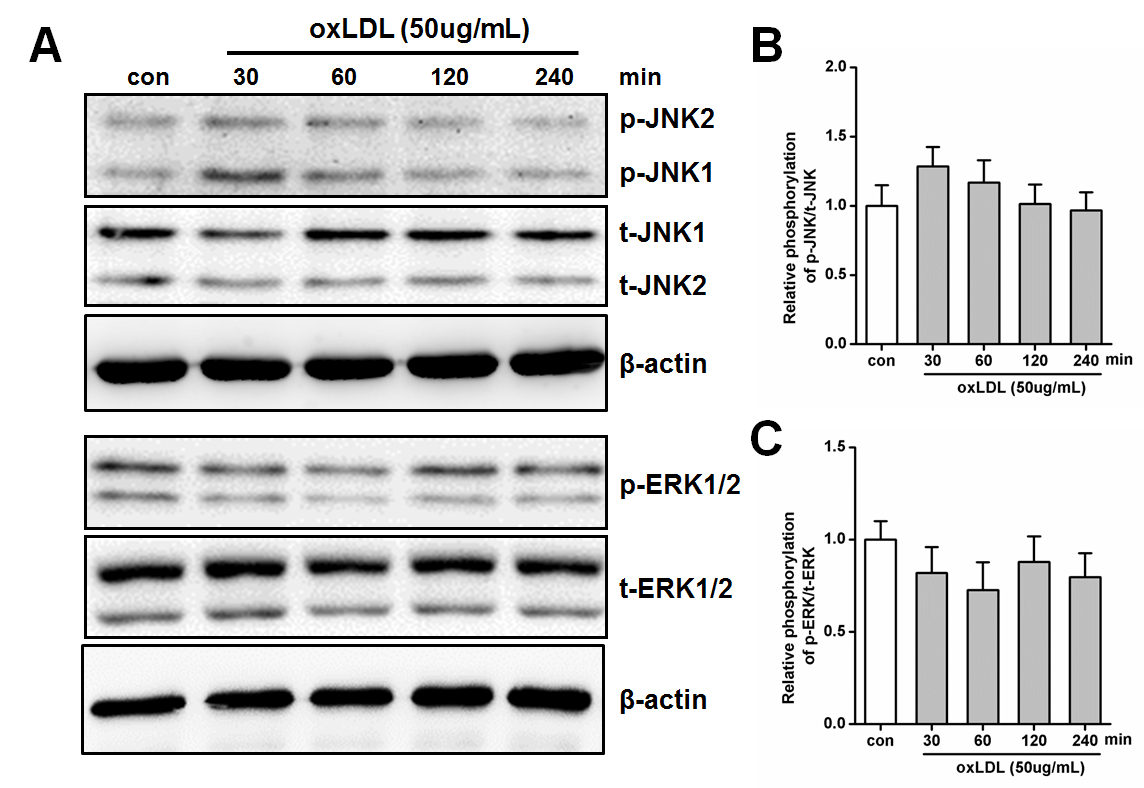

Supplement: Figure S3 — oxLDL has none effect on ERK1/2 and JNK in SMCs. (A)–(C) SMCs were incubated for increasing amount of time (30, 60,120 and 240 min with 50 ug/mL)of oxLDL. The phosphorylation of JNK and ERK1/2 was detected by Western blotting (A) and quantified by densitometry in 3 independent experiments (B and C) as relative units (JNK or ERK1/2 phosphorylated protein/total protein). (Mean ± SD, n = 3). (TIF) [file pone.0095935.s003.tif]

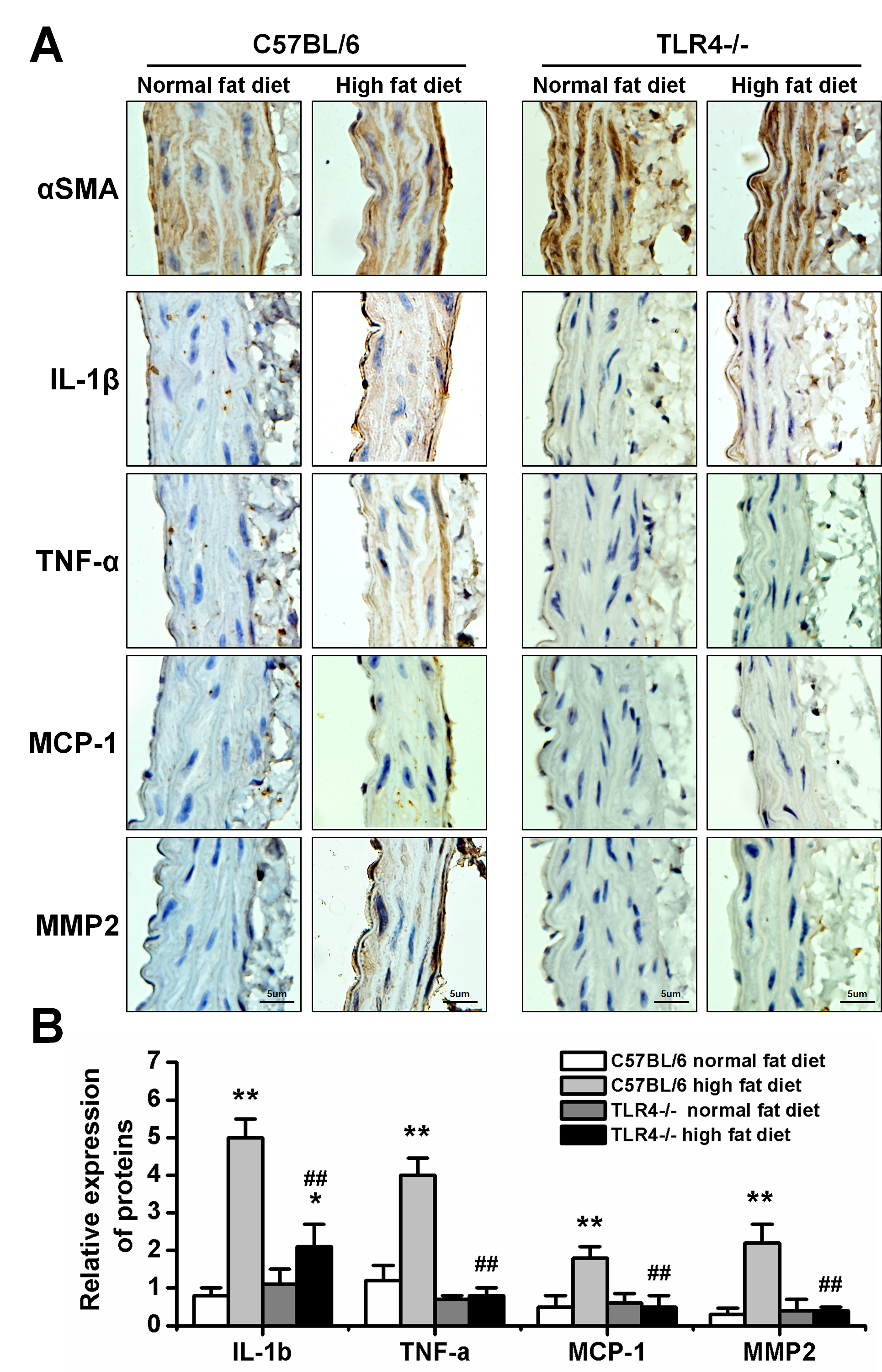

Supplement: Figure S4 — The expression of cytokines in the SMCs of artery of wild type (C57BL/6) and TLR4−/− mice fed with high fat or normal fat diet. The wild type and TLR4−/− mice fed with high fat or normal fat diet for one month. (A) The αSMA had been used to identify the SMCs. In the αSMA-positive region, the expression levels of IL-1β, TNF-α, MCP-1 and MMP-2 detected by IHC were determined by assessing its staining using software image pro-plus 6.0. (B) The results were showed as integrated optical density (IOD)/area. Three different sections and five different fields in each section have been detected. (n = 3, Mean±SD, *P<0.05, **P<0.01 compared with normal fat diet fed group; ##P<0.01 compared with C57BL/6 group). (TIF) [file pone.0095935.s004.tif]

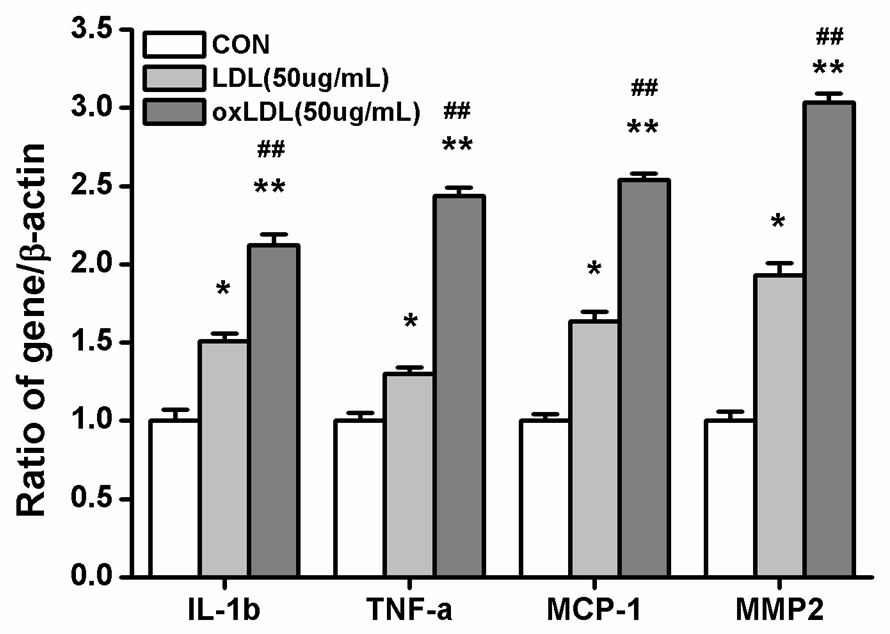

Supplement: Figure S5 — LDL and oxLDL regulated IL-1β, TNF-α, MCP-1 and MMP-2 expression. After LDL (50 ug/mL) or oxLDL (50 ug/mL) incubated with SMCs for 48 hours, expression of IL-1β, TNF-α, MCP-1 and MMP-2 had been test by Realtime-PCR. Un-treatment of LDL or oxLDL were used as control and normalized with β-actin. (Mean ± SD, n = 3, *P<0.05, **P<0.01 compared with CON; ##P<0.01 compared with LDL treatment group). (TIF) [file pone.0095935.s005.tif]
